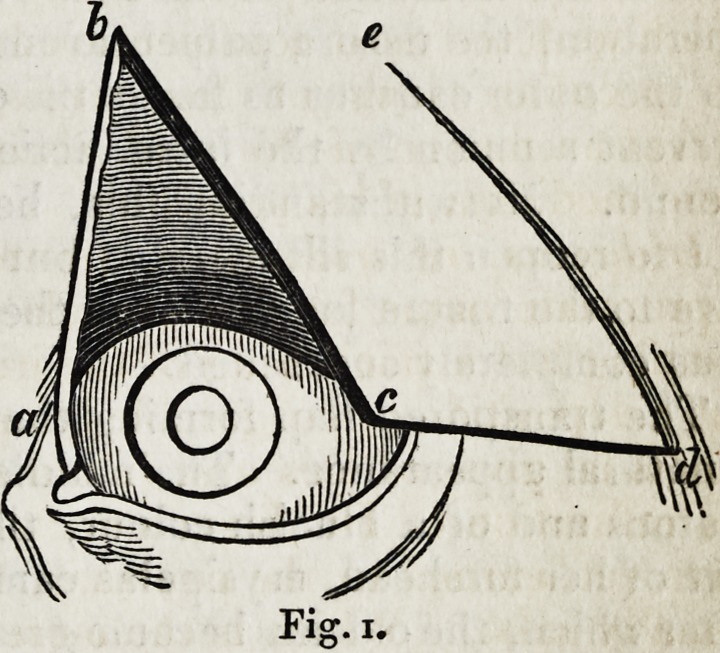# A Journal of Ophthalmology

**Published:** 1837-10

**Authors:** 


### Art. XIV.

*Zeitschrift für die Ophthalmologie.* Herausgegeben von Dr. F. A. v. Ammon. Fünften Bandes; Zweiter und Dritter Heft.—*Heidelberg und Leipzig,* 1836.

*A Journal of Ophthalmology.* Edited by Dr. Ammon, of Dresden. Fifth Volume; second and third Numbers in one.—8vo. pp. 287; two Plates. *Heidelberg and Leipsic,* 1836.

We have already expressed our very favorable opinion of this Journal, which continues to be conducted with all its pristine spirit and acumen. The present double Number contains six extended communications, no fewer than forty miscellaneous extracts, and short notices of thirteen new publications. The extended communications are, 1. The Eye, as an object of Medical Police, by Dr. Beger; 2. A Case of Amaurosis, with the dissection, and Remarks on Amaurosis attended with distinct organic changes, by Dr. Beck; 3. A continuation of Researches on the Anatomy and Pathology of the Eyelids, by Dr. Zeis; 4. An Ophthalmological Tour to Vienna and Prague, in 1834, by Dr. Thune, of Copenhagen; 5. On a peculiar Degeneration of the Iris, consequent to Iritis, by Dr. Klemmer; 6. Formation of an upper Eyelid from the Integuments of the Temple, with Restoration also of the lower Eyelid, by Dr. Ammon.

We shall give a brief account of the more important memoirs.

1. Dr. Beger introduces his subject by some eloquent remarks on the importance of the eye in regard to human industry and happiness, and it is pleasing to observe the correctness with which he keeps these objects in view through the whole of his elaborate essay. That it is an imperative duty of statesmen, physicians, and indeed men of every rank, to lend their aid in preserving sight, is the point which he labours to establish. He divides his essay into three sections, and each section into several chapters; and it may amuse our readers to run over the heads of discourse of the worthy German.

His *first* section comprehends the precautions to be observed regarding those persons who are to be allowed to practise as oculists. Under this head, he discusses institutions for the education of the oculist, examinations of candidates in this department, the prevention of eye-quackery, popular works on eye-diseases, and the censure* of ophthalmological publications. His *second* section embraces the means of preventing eye-diseases. Under this head he considers the injurious construction of dwelling-houses and places of public resort; the cleaning and paving of streets, so as to lessen the quantity of dust; the lighting of the streets and public places; the injurious influence of artificial drinks, as wine, beer, &c.; the bad effects of tobacco, snuff, and cosmetics; the effects of improper clothing; the influence of trades and employments; the effects of glasses, and the propriety of restraining the sale of lead spectacles; and the means of preventing and guarding against epidemic and contagious diseases of the eye. His *third* section relates to public oph-

* This term will probably sound but harshly to English ears. What would some of our so-called oculists think, were a law passed that any one publishing such a statement as that he could cure cataract by touching it with a hair pencil dipped in a solution of caustic potash, should have his ears nailed to the pillory!

thalmic institutions, including eye-infirmaries and dispensaries, hospitals or asylums, schools of industry and seminaries for the blind. On all these various topics, Dr. Beger displays a great deal of accurate information and a truly philanthropic spirit.

2. Dr. Beck's *Case of Amaurosis* from atrophy of the optic nerves, occasioned by the pressure of ossified and dilated internal carotid arteries, is interesting, and we shall transfer it to our Selections. The remarks by which it is accompanied, referring to a great number of recorded cases of amaurosis, are good, though rather rambling. The idea has often occurred to us that a *codex casuisticus* of this disease,—that is, a collection of all the best cases on record, particularly of those with dissections, arranged in systematic order,—would prove a valuable addition to ophthalmological literature. We wish some laborious German would think of it.

3. Dr. Zeis, of Dresden, had previously published, in the fourth volume of Dr. Ammon's Journal, a learned and laborious paper on the *Structure of the Eyelids, and especially on the Meibomian follicles.* The present paper follows up his former researches, and we shall give a condensed view of the whole in our next Number.

Dr. Thune communicates an amusing sketch of the practice of Drs. Jäger and Rosas, the two great rival oculists of Vienna, and of that of Dr. Fischer, of Prague; all three pupils of Beer.

Dr. Fischer is rather an elderly man, who, with only one eye, and that one aided by a glass, operates with great expertness and eminent success. In the Reports of his Clinic, he has described several curious irregularities of the lacrymo-nasal canal. He has in his possession preparations of a complete closure of its inferior aperture, and of an expansion of the lower portion of it into a cul-de-sac, in the side of which is the communication with the nostril. Among his preparations is one of ossification of the retina and choroid, extending as far forwards as the attachment of the iris. The ossification corresponds exactly to the globular figure of the eye, and its inner surface is studded with minute irregular exostoses.

In forming an artificial pupil by excision, in individuals the height of whose nose prevents the use of the common instruments for laying hold of the iris, he employs a very small pair of forceps, an inch long, and somewhat bent, with which he easily lays hold of the iris, and, drawing a portion of it through the wound of the cornea, snips it off. In general he uses Beer's instruments, only somewhat reduced in size. He has performed extraction innumerable times, as well as operations for forming an artificial pupil. It could scarcely be otherwise; since almost all his patients are treated and boarded gratis. He possesses hundreds of lenses which he has extracted, and is thus able to shew his pupils the remarkable differences they present in form, colour, size, consistence, &c.

A chronic conjunctivitis prevailed for several years in Prague, affecting particularly the conjunctiva of the upper eyelid, and ending in pannus, especially among the poor, who but too often neglected the disease in its early stage. Fischer tried various means for removing the granular state of the lids in this disease, without effect. Touching the diseased surface with nitrate of silver seemed rather to favour the progress of the symptom in question than abate it. At the time of Dr.

Thune's visit, he employed a salve composed of from half-a-grain to three grains of white precipitate of mercury and a drachm of lard, which he pencilled pretty freely on the inner surface of the eyelids and over the eyeball, twice a day. Internal remedies and counter-irritation he considered useless in this disease; and indeed he seldom employs blisters in any case.

5. Those at all conversant with the diseases of the eye must frequently have observed the peculiar state of the iris consequent to iritis, which Dr. Jäger, of Vienna, designates by the name of *Staphyloma iridis*, and to which Dr. Klemmer proposes to apply the new appellation of *Iridoncosis*. The anterior surface of the iris, in the diseased condition to which these names are applied, has lost its natural colour, is often blackish, or even presents a hue so deeply black that we might suppose the iris to have been absorbed at the part affected, or that a piece of it had been cut out for the formation of an artificial pupil.

Dr. Jäger's pathology of this state of the iris is very different from that of Klemmer's. Both are agreed that it is one of the many sequelæ of chronic, and generally of some specific, iritis. In consequence of inflammation, Dr. Jäger thinks the iris loses its natural firmness of texture, and becomes preternaturally adherent to the subjacent uvea. Next, he believes the aqueous humour of the posterior chamber presses the uvea forwards through the attenuated iris, and that thus the staphyloma iridis* is formed. Dr. Jäger has not himself published on the subject; but, in the account of his doctrine furnished us by his pupil, Dr. Froriep,† and referred to by Dr. Klemmer, we have observed no notice of the influence which the contracted and adherent state of the pupil is likely to have in the cases in question, in promoting the pressure forwards of the uvea by the aqueous humour, in consequence of this fluid not being permitted to flow in what is generally regarded its natural course, namely, through the pupil into the anterior chamber.

That, in such cases as Dr. Jäger designates by the name of *Staphylomata iridis*, the iris is not actually absorbed in its whole thickness, is proven from the want of sight, and from the fact that, under the circumstances in question, he has formed an artificial pupil with success.

In ten deadly chapters, occupying forty-nine pages, every word of which we have most conscientiously perused, does Dr. Klemmer open up his peculiar views of *Iridoncosis*, setting altogether at defiance the Terentian maxim of *Ne quid nimis*. We must content ourselves with a mere modicum of his superflux.

*Iridoncus*, or *iridoncosis*, (from ἶρις and ὄγκος,) he proposes as the appropriate name for this sequela of iritis, which he contends is not a thinning but a thickening of the iris; not a shining through of the uvea, but a deposition of coagulable lymph in the parenchyma of the iris. The proofs he gives of this are, in our opinion, imperfect; and no less so is his refutation of Dr. Jäger's doctrine on the subject. For anything we have yet learned to the contrary, both conditions of the iris may occasionally exist. Which is the more perfect, and by what marks they are to be distinguished, must be left for future enquirers to determine, and

* *Staphyloma uveæ* would be more correct, and would serve to distinguish this disease from a protrusion of the iris through the cornea.

† De Corneitide Scrofulosa, p. 9. Jenæ, 1830.

especially for those who shall have opportunities of dissecting eyes affected with the consequences of iritis.

Sometimes the black discoloration in cases of staphyloma iridis or iridoncosis exists merely in small insulated points; in other instances the whole iris is affected, except towards the pupil, where the iris is more apt to preserve its natural texture. Sometimes the black colour is present only close to the great circumference of the iris, and forms a complete ring; in other cases it presents a triangular form, the basis of the triangle being turned towards the ciliary, and the apex towards the pupillary edge of the iris. The disease may be total or partial; its surface undulatory or uniform; its colour is not always black, but is sometimes grey or bluish white, or blackish blue; and the spot affected often presents a striated appearance, from the vessels or nerves passing through it.

Dr. Klemmer relates only one dissection, and that not of the human eye, but of the eye of an ox. His magnified figures of the disease are good.

6. The *Formation of a new lower Eyelid* has succeeded in the hands of several operators, as well as in those of Dieffenbach; but we have here the first account of an upper eyelid, supplied from the integuments of the temple; an operation very creditable to the talents of Dr. Ammon.

Mrs. S. had the misfortune to have her face sadly disfigured by syphilis. She lost her nose; her upper lip was so much shortened, that she could not cover the teeth of the upper jaw; the left upper eyelid was destroyed, and the lower in a state of complete ectropium. Several extensive cicatrices on the hairy scalp and on the forehead shewed the previous existence of necrosis, with exfoliations of the outer table of the skull. A considerable portion of the upper, outer, and lower edge of the orbit had been lost in this way. The greater part of the left upper eyelid was so completely removed by ulceration, that its remains surrounded merely, without covering, the eyeball. The conjunctiva of the small portion which remained was turned outwards, and its tarsal edge very irregular.

Dr. Ammon began his operation by insulating and separating from the temple the flap of skin (Fig. I. *b, c, d, e,*) by which the defective upper eye-

Fig. II.

Fig. I.

lid was to be supplied; he then divided all the adhesions of the old eyelid, and prepared the place (Fig. I. *a, b, c,*) for the reception of the new one. He formed the flap by a horizontal incision (Fig. I. *c, d,*) two inches and a half in length, to which he joined the perpendicular one, (Fig. I. *d, e,*) and then dissected it off. He returned the shrunken remains of the old eyelid with the bistoury; but unfortunately found it impossible to separate enough of conjunctiva from it to form a lining membrane for the new eyelid.

As soon as the bleeding had ceased, the flap forming the new eyelid having been brought into such a position that it covered the eye, it was secured along its inner edge (Fig. II. *b, c,*) by Dieffenbach's suture; and thus ended the formation of the upper eyelid.

To remedy the ectropium of the lower eyelid, Dr. Ammon first of all carried an incision through the skin, parallel to its edge, and then dissected it from its unnatural adhesions; he next extirpated a horizontal fold of the exuberant conjunctiva; and, lastly, having made a cut, like a button-hole, through the eyelid, about four lines from its edge, by means of a ligature he laid hold of that part of the conjunctiva which still remained attached to the tarsal portion of the eyelid, drew out this ligature through the wound, and so fixed the lid in its natural position.

At the temporal angle, the upper and lower eyelids were now connected by the twisted suture, which after some hours, however, was removed; Dr. Ammon fearing that thereby the fissura palpebrarum might be made too small. The wound on the temple, caused by the transplantation of the new eyelid, was covered with charpie and a thick compress wet with water.

Next day the transplanted skin was somewhat swollen, so much so that the fissura palpebrarum was no longer visible, and the eyeball was entirely concealed. By injecting tepid water, Dr. Ammon removed the matter which collected on the eye; but, notwithstanding this precaution, a considerable œdema took place of the conjunctiva, which rendered the injections still more necessary. The union of the inner edge of the transplanted flap did not take place entirely by the first intention, so that, as the stitches were gradually withdrawn, stripes of sticking plaster were applied. The wound on the temple granulated favorably. The cut through the lower eyelid, into which the conjunctiva had been drawn, closed perfectly; so that the eyelid, after the œdema had subsided, maintained its proper position.

The granulation of the wound on the temple proceeded, and along with it the formation of the new outer canthus. Three weeks after this operation, the fissura palpebrarum appearing too small, Dr. Ammon slit up the outer canthus as far as the edge of the orbit, and endeavoured to prevent reunion by the introduction of charpie between the lips of the wound. Notwithstanding this, he was obliged, two months afterwards, not to reopen this slit merely, but to extirpate a stripe of skin, so as to give to the fissura palpebrarum the proper degree of length; in which he thus completely succeeded.

The transposed flap forming the upper eyelid assumed more and more a natural appearance. The middle of it, however, continued to be œdematous and of a blueish colour, till, on forming a new nose for Mrs. S. out of her forehead, erysipelas came on, and spread to the new eyelid; after which, the œdema became greatly less, and at last vanished entirely.

Seven months after its formation, the new eyelid closed over the eyeball, without irritating it; it could be lifted from it like a natural eyelid, but generally it hung over it in a state of semiptosis. The cicatrix on the temple was very small, so that it was difficult to believe that so considerable a portion of the integuments had been removed from that part.

## Figures and Tables

**Fig. II. f1:**
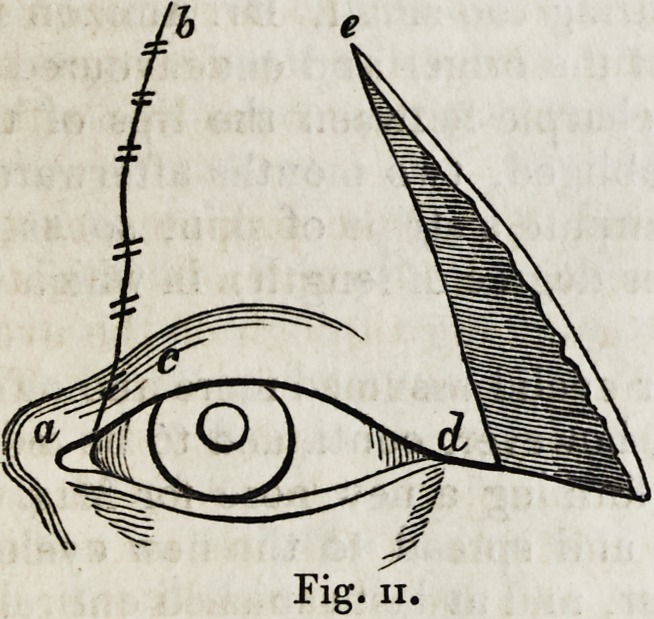


**Fig. I. f2:**